# Metabolomic Alterations in the Tear Fluids of Patients With Superior Limbic Keratoconjunctivitis

**DOI:** 10.3389/fmed.2021.797630

**Published:** 2022-01-18

**Authors:** Yan Zong, Chao Cheng, Kunke Li, Ran Xue, Ziyan Chen, Xiuping Liu, Kaili Wu

**Affiliations:** Zhongshan Ophthalmic Center, State Key Laboratory of Ophthalmology, Sun Yat-sen University, Guangdong Provincial Clinical Research Center for Ocular Diseases, Guangzhou, China

**Keywords:** metabolomics, tear fluids, superior limbic keratoconjunctivitis, liquid chromatography with tandem mass spectrometry (LC-MS/MS), metabolites

## Abstract

**Purpose:**

Superior limbic keratoconjunctivitis (SLK) is a bilateral, chronic inflammatory disease that recurs for up to several years; however, the fundamental processes involved in its pathogenic mechanisms remain unknown. We aimed to investigate the metabolomic alterations in the tear fluids of patients with superior limbic keratoconjunctivitis (SLK) compared with those of healthy volunteers (Ctrl group).

**Methods:**

We performed a cross-sectional study involving 42 subjects. Tear fluid was taken from one eye of 24 SLK patients (40.13 ± 14.55 years, 83.33% female) and 18 healthy volunteers (Ctrl, 39.89 ± 9.2 years, 72.22% female) using Schirmer strips. After the liquid extraction of tear metabolites, samples were infused into the QE HFX Orbitrap mass spectrometer in both positive and negative ion mode. Metabolites were quantitatively analyzed and matched with entries in the HMDB database. Metabolic differences between the SLK group and the control group were identified based on multivariate statistical analysis. Open database sources, including SMPDB and MetaboAnalyst, were used to identify metabolic pathways.

**Results:**

Among 179 metabolites retained for annotation, 133 metabolites were finally identified, among which 50 were found to be significantly changed in SLK patients. Of these 50 metabolites, 31 metabolites significantly increased and 19 metabolites decreased in SLK patients. The altered metabolites are mainly involved in α linolenic acid and linoleic acid metabolism, ketone body metabolism, butyrate metabolism, mitochondrial electron transport chain, carnitine synthesis, and so on. The most significantly changed pathway was linoleic acid metabolism. To explore the utility of tear biomarkers, a model combining 9 metabolites (phenol, ethyl glucuronide, eicosapentaenoic acid, 12-keto-leukotriene B4, linoleic acid, hypoxanthine, triethanolamine, 1-nitrohexane, and terephthalic acid) was selected as a candidate biomarker.

**Conclusion:**

The results reveal that SLK has a specific metabolomic profile, of which some key elements can serve as potential biomarkers of SLK for diagnostic and prognostic purposes. The findings of this study are novel and provide a basis for further investigations of the mechanism of SLK.

## Introduction

Superior limbic keratoconjunctivitis (SLK) is a bilateral, chronic inflammatory disease that affects the upper bulbar conjunctiva, superior limbus and adjacent cornea (1, 2). Sixty years have passed since SLK was first described by Theodore in 1961, and increasing reports have revealed the association of SLK with other diseases, such as dry eye disease (DED) (3), thyroid disease (4), conjunctivochalasis (2), and so on. Although there have been some aetiological hypotheses and meaningful explorations, the exact pathogenesis of SLK is unknown. SLK has been characterized by accumulations of mast cells, inflammatory mediators (including stem cell factor, thymic stromal lymphopoietin and matrix metalloproteinases), and tear cytokines (such as monocyte chemoattractant protein-1), which are arousing the interest of researchers (5–7). However, targeted treatment based on these hypotheses has poor effects, and the aetiological factors and mechanisms of the disease are still unknown.

Tear fluid is composed of proteins, carbohydrates, lipids, electrolytes, and some small organic molecules, which play great roles in protecting and maintaining ocular surface normality. In turn, molecular alterations of tear fluids would provide a great deal of molecular information that is useful for the diagnosis, prognosis, and treatment of ocular surface diseases (8–11). Investigation at the molecular level is the most direct approach to exploring the development mechanism and providing reliable evidence. In recent years, exploring disease biomarkers from tears has been of increased interest for many researchers, especially by characterizing the proteomic and metabolomic changes in the tears of different diseases (12–16).

Metabolomics is focused on a comprehensive analysis of metabolites in a biological system and metabolic changes in response to pathophysiological stimuli, genetic modifications and/or environmental perturbations (12). The main analytical techniques adopted are nuclear magnetic resonance (NMR) spectroscopy, gas chromatography-tandem mass spectrometry (GC–MS), and liquid chromatography-tandem mass spectrometry (LC–MS/MS). Metabolomics has been widely used to assess biological systems, providing molecular information related to phenotypes since metabolites are the ultimate product of gene, mRNA and protein activity (17). Moreover, in terms of the high-throughput profiling of the whole metabolome in a disease condition, identifying biomarkers becoming possible (18, 19). With advances in technology, untargeted LC–MS metabolomic analysis of tear fluid has been applied in clinical and animal studies of several eye diseases, such as keratoconus (12, 15, 20), dry eye (13, 21), and multiple sclerosis (16). Studies of metabolomics used in eye diseases have been summarized in a recent review article (12). As the reviewers suggested, to date, possible biomarker candidates for dry eye disease are lipid metabolites and androgens, and those for keratoconus are cytokeratins, urea, citrate cycle, and oxidative stress metabolites. In addition, palmitoylcarnitine, sphingolipids, vitamin-D-related metabolites, and steroid precursors may be related to glaucoma, and the dysregulation of amino acid and carnitine metabolism is critical in the development and progression of diabetic retinopathy (12).

In the present study, we analyzed tear samples obtained from a clinical cohort of 42 subjects and investigated the tear metabolomic differences between SLK patients and healthy controls. We aimed to identify the metabolites in tears that are pathologically relevant to SLK. In addition, we constructed a potential metabolite biomarker model to assist us in distinguishing SLK from a healthy status.

## Materials and Methods

### Study Population

A total of 24 SLK patients and 18 healthy subjects were recruited from October 2020 to March 2021 at Zhongshan Ophthalmic Center, a tertiary eye hospital at Sun Yat-Sen University in Guangzhou, China. All subjects were selected to obtain age- and sex-matched study cohorts. SLK patients were diagnosed according to the following criteria: (1) fluorescein staining at the superior limbus and adjacent conjunctiva above the limbus; (2) superior bulbar conjunctiva hyperaemia and/or conjunctivochalasis; (3) papillae and hypertrophy in the tarsal conjunctiva of the upper lid; and (4) punctate or confluent cornea staining (2). Exclusion criteria included any of the following conditions: (1) rheumatism, dry syndrome, or other diseases affecting tear secretion; (2) a history of eye topical therapy or use of contact lenses within the previous 3 months; or (3) ocular diseases within the previous 6 months. Both eyes were evaluated, but one eye per subject was randomly chosen for statistical analysis. The study was conducted according to the Declaration of Helsinki (World Medical Association, 2013) and approved by the Institutional Review Board of the Zhongshan Ophthalmic Center. All patients were informed about the procedures and provided written informed consent to participate in the study. Basic demographic information, such as age, sex, and medical history, was recorded. Ocular surface examination for every subject included visual acuity, intraocular pressure, slit lamp examination, tear breakup time (TBUT), corneal staining and Schirmer I test (SIT). The clinical and demographic features of the enrolled subjects are summarized in Table 1.

**Table 1:** Demographic characteristics of study subjects in the SLK and Ctrl groups.

**Feature**	**Ctrl**	**SLK**	***p* value**
	**(*n* = 18)**	**(*n* = 24)**	
Sex (# female, %)	13, 72.22%	20, 83.33%	0.46
Age (years)	39.89 ± 9.24	40.13 ± 14.55	0.96
FBUT (S)	8.28 ± 2.02	3.88 ± 2.35	0.00
SIT (mm)	7.5 (4.5)	6.0 (8.63)	0.37
Tear volume (μl)	5.6 (4.38)	4.90 (7.7)	0.22

*FBUT, fluorescein breakup time; SIT, Schirmer I Test; p value less than 0.05 was considered statistically significant*.

### Sample Collection

Tear samples were collected by Schirmer strips (Tianjin Jingming New Technological Development Co., Ltd, China) as we reported previously (22). The Schirmer strip was placed over the temporal one-third of the lower eyelid for 5 min. Then, the strip was removed from the eye, and the length of the moistened area was measured using the millimeter scale on the strip. After the SIT, every filter strip was placed in a single 1.5 mL microtube (Axygen®, Jiangsu, China) and stored at −80°C.

### Metabolite Extraction and LC–MS/MS Analysis

The parts of Schirmer strips that were imbibed by tears were cut into 2–3 mm paper pieces and transferred into GV 0.22 μm, Ultrafree®-MC Filter Devices (Merck Millipore Ltd, Ireland). Each filter cup was prefilled with ultra-pure grade water 20 times the volume of tears in Schirmer strips [calculated from 7 μl tears/10 mm wetted Schirmer test paper (23)]. Each device was placed vertically on an ice box for 30 min and then centrifuged at 13,800 × g for 15 min at 4°C. Then, 100 μl of the filtrate was transferred to an EP tube. After the addition of 400 μl of extract solution (acetonitrile: methanol = 1:1, containing isotopically labeled internal standard mixture), the samples were vortexed for 30 s, sonicated for 10 min in an ice-water bath, and incubated for 1 h at −40°C to precipitate proteins. Then, the sample was centrifuged at 13,800 × g for 15 min at 4°C. The resulting supernatant was transferred to a fresh glass vial for analysis. The quality control (QC) sample was prepared by mixing an equal aliquot of the supernatants from all of the samples. Seven QC samples were injected in a random order.

LC–MS/MS analyses were performed with an integrated platform (Guangdong Magigene Biotechnology Co., Ltd. Guangzhou, China), using an ultra-high performance liquid chromatography (UHPLC) system (Vanquish, Thermo Fisher Scientific) with a UPLC BEH Amide column (2.1 mm × 100 mm, 1.7 μm) coupled to a Q Exactive HFX mass spectrometer (Orbitrap MS, Thermo). The mobile phase consisted of 25 mmol/l ammonium acetate and 25 mmol/l ammonia hydroxide in water (pH = 9.75) (A) and acetonitrile (B). The autosampler temperature was 4°C, and the injection volume was 2 μl.

A QE HFX mass spectrometer was used for its ability to acquire MS/MS spectra in information-dependent acquisition (IDA) mode in the control of the acquisition software (Xcalibur, Thermo). In this mode, the acquisition software continuously evaluates the full-scan MS spectrum. The electrospray ionization (ESI) source conditions were set as follows: a sheath gas flow rate of 30 Arb, Aux gas flow rate of 25 Arb, capillary temperature of 350°C, full MS resolution of 60,000, MS/MS resolution of 7500, collision energy of 10/30/60 in NCE mode, and spray voltage of 3.6 kV (positive) or −3.2 kV (negative).

### Data Processing and the Identification of Metabolites

The raw data were converted to the mzXML format using ProteoWizard and processed with an in-house program, which was developed using R and based on XCMS, for peak detection, extraction, alignment, and integration. MS/MS spectra of 6 metabolites were provided in the Supplementary Materials as representative spectra (Supplementary Figure 1). The metabolites were identified by matching MS/MS spectra with an in-house MS2 database. The cut-off for annotation was set at 0.3. All identified metabolites were matched with entries in the Human Metabolome Database (HMDB, http://www.hmdb.ca), Kyoto Encyclopedia of Genes and Genomes (KEGG, http://www.kegg.jp) and Small Molecule Pathway Database (SMPDB, https://smpdb.ca/).

### Metabolomics and Statistical Analysis

All statistical analyses were performed using SPSS software (version 26.0, IBM, Armonk, NY, USA). Normality tests to numerical data were applied first. The results were expressed as the mean ± standard deviation or median (interquartile range) for numerical variables and as the number (percent) for categorical variables. The comparison of the mean of numerical variables between the groups was assessed using Student's *t*-test or Mann-Whitney *U*-test as appropriate. For categorical variables, differences between groups were analyzed using Fisher's exact test. *P*-values less than 0.05 were considered statistically significant.

All metabolites were normalized by log2 transformation before analysis. Principal component analysis (PCA), orthogonal partial least squares discriminant analysis (OPLS-DA) and volcano plotting were performed to reveal the data structure and identify metabolic differences in Simca-P V.14.1 (Umetrics AB). Metabolites with (1) an MS2 score of >0.8, (2) a variable importance in the projection (VIP) of >1, and (3) a *P* of < 0.05 (*t*-test) were regarded as differentially abundant. GraphPad Prism 8 (GraphPad Software, Inc., 2018, La Jolla, CA, USA) was used to plot the box-and-whisker plots. MetaboAnalyst 5.0 (http://www.metaboanalyst.ca) was used to perform pathway topology analysis and generate operating characteristic curves (ROCs) (24), aiming to identify the most relevant metabolic pathways and assess the potential combined biomarker model.

## Results

### Demographic Data and Ocular Surface Parameters

A total of 42 subjects were enrolled in this study, with 24 patients in the SLK group and 18 volunteers in the healthy control group (Ctrl). The demographic data and ocular surface parameters are shown in Table 1. The sex proportions and ages showed no significant difference between groups (*P* = 0.398 and *P* = 0.955, respectively). The patients in the SLK group showed lower values of fluorescein breakup time (FBUT) than those in the healthy control group (*P* = 0.000). Considering challenges in metabolite extraction and instrumental sensitivity (25), only subjects with appropriate Schirmer I test (SIT) scores were included in the study, so there was no difference in SIT scores or tear volume in Schirmer strips between the two groups (*P* = 0.727 and *P* = 0.515, respectively).

### Overall Metabolites of Tear Samples

Through data processing and identification of MS peaks, a total of 179 metabolites were retained with a cut-off of 0.3 for annotation, including 99 metabolites derived from positive ionization modes and 89 metabolites derived from negative ionization modes. Ensuring the confidence of annotated metabolites, 133 metabolites, including 71 metabolites in positive ion mode and 62 metabolites in negative ion mode, with an MS2 score of >0.8 were finally identified in tears in the study. These metabolites were selected for further analysis, with part having no precedence reports in the literature (8, 26).

### Metabolomic Profiling Differences Between the SLK and Ctrl Groups

**Figure 1:**
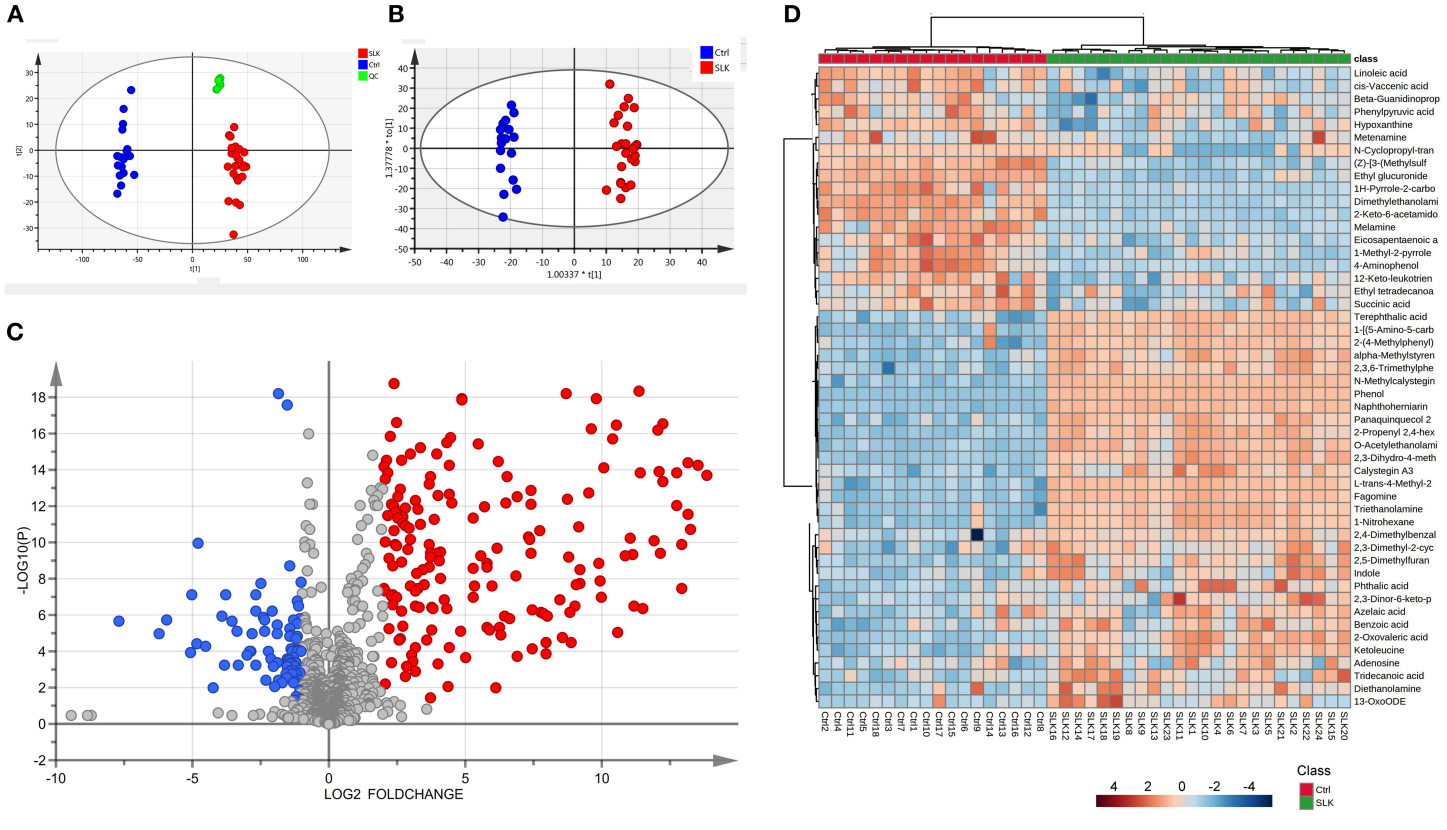
Metabolomics analysis of tear samples from patients with superior limbic keratoconjunctivitis (SLK) compared with the controls (Ctrl). **(A)** Principal component analysis (PCA) score plots of Ctrl (blue), SLK (red), and quality control (QC, green). The X-axis (t[1]) and Y-axis (t[2]) indicate the first and second principal components, respectively. **(B)** Orthogonal projections to latent structures-discriminate analysis (OPLS-DA) score plots for the Ctrl (blue) and SLK (red) groups. The X-axis (t[1]) and Y-axis (to[1]) indicate the predictive and orthogonal directions, respectively. **(C)** Volcano plots highlighting the tear metabolites that increased (red) or decreased (blue) in SLK tear fluids compared to the control group, with *p* < 0.05, log_2_FC >1 or < -1. **(D)** Hierarchical cluster analysis and heatmap of the differentially expressed metabolites from the SLK and Ctrl groups. The color code in the heatmap represents the relative metabolite abundance: red and blue colors indicate increased and decreased levels of each metabolite in the SLK group versus the Ctrl group, respectively.

The metabolomic profiles of the two groups were first compared by means of principal component analysis (PCA), and for exploring the stability and reliability of the data, QC samples were contained. As shown in Figure 1A, PCA revealed the internal structure of the data and indicated a distinct separation among the SLK, Ctrl, and QC groups. The QC samples clustered tightly together indicating high quality of the data. The samples were within the 95% confidence interval (CI). Furthermore, OPLS-DA showed more reliable differential metabolite information between SLK and Ctrl tears (Figure 1B). All the samples were within the 95% CI. On the basis of volcano plots, 31 metabolites were significantly increased and 19 were decreased in the SLK group compared with the control group (Figure 1C, *p* < 0.05, log2FC > 1 or < -1, VIP > 1). To further understand the alterations in metabolites between the two groups, we used a heatmap to visually represent the differentially abundant metabolites. The results showed correct sample group clustering and the dendrogram structure by Euclidean distance and resulted in two main clusters, relating to the compared samples of the SLK and Ctrl groups (Figure 1D). These data analyses confirmed the existence of significant differences in the metabolic profile between the two groups.

**Figure 2:**
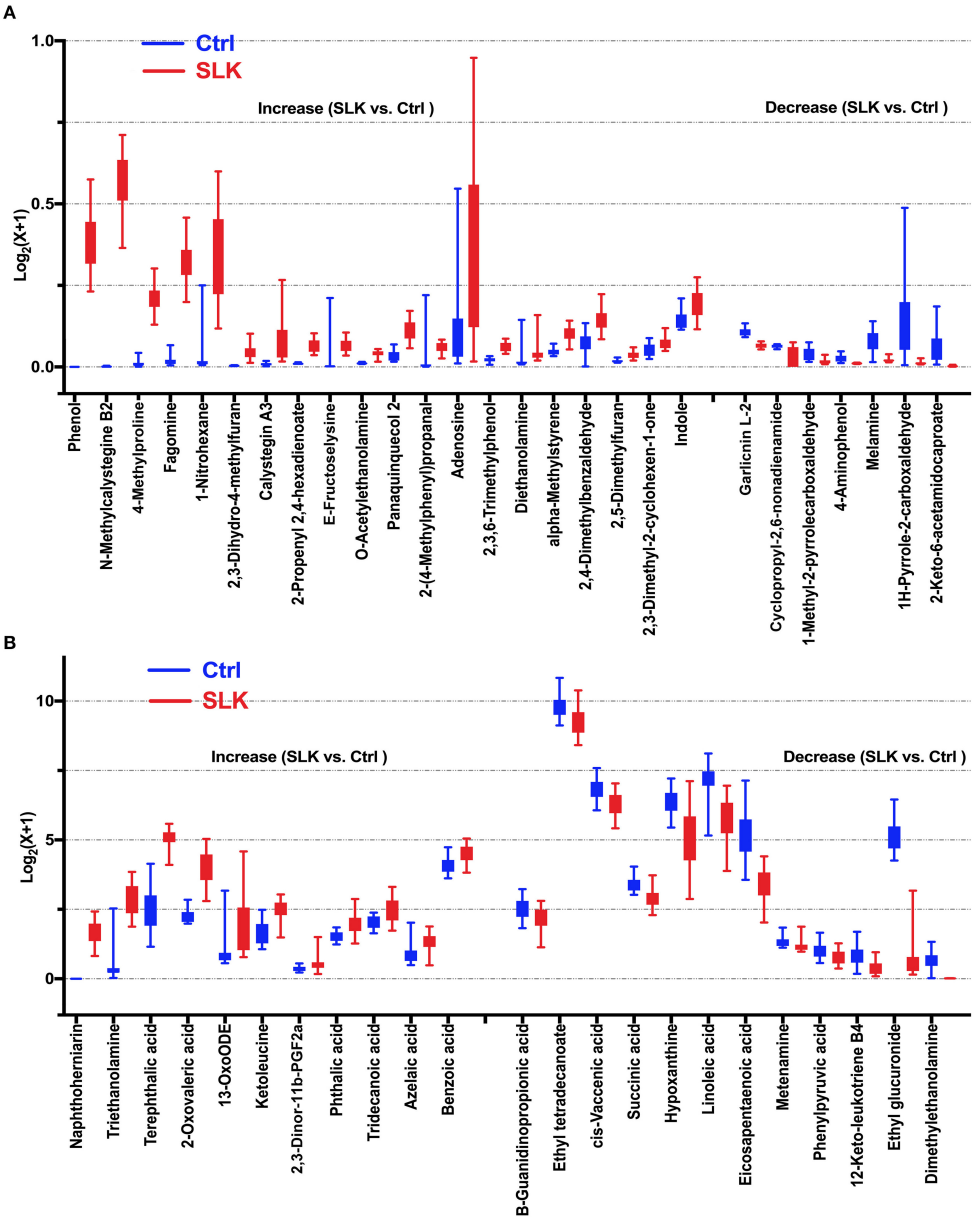
Boxplots show significantly altered metabolites in the tears of SLK patients. Compared with Control subjects, 19 decreased metabolites and 31 increased metabolites represented by relative expression in the boxplot (SLK vs. Ctrl: *P* < 0.05). Specifically, for better visualization of box plots in this analysis, the expressions of the metabolites was converted into log2(x + 1) values and a different Y-axis scales and range were applied in **(A,B)**. **(A)** Metabolites with maximum converted values less than 1 [log2(x + 1) ≤ 1], **(B)** Metabolites with maximum converted values more than 1 [log2(x + 1) > 1]. Both figures present increased metabolites in the left [20 in **(A)**, 11 in **(B)**] and decreased metabolites in the right [7 in **(A)**, 12 in **(B)**], separated by two tick intervals.

Using the model that was optimized at 2 principal components, with R^2^Y = 0.986 and Q^2^ = 0.961, we employed the VIP score to help select differential metabolites because higher values of VIP indicate metabolites that are more important to classification (Supplementary Table 1). Combined with the VIP and *P*-values, we identified 50 differential metabolites that changed significantly, contributing to the separation, 32 metabolites in positive ion mode and 18 metabolites in negative ion mode (Figure 2). According to the fold change, 31 metabolites were significantly increased in SLK patients. In contrast, the levels of the other 19 metabolites were significantly decreased in the SLK group.

### Metabolite Set Enrichment and Pathway Analysis

**Figure 3:**
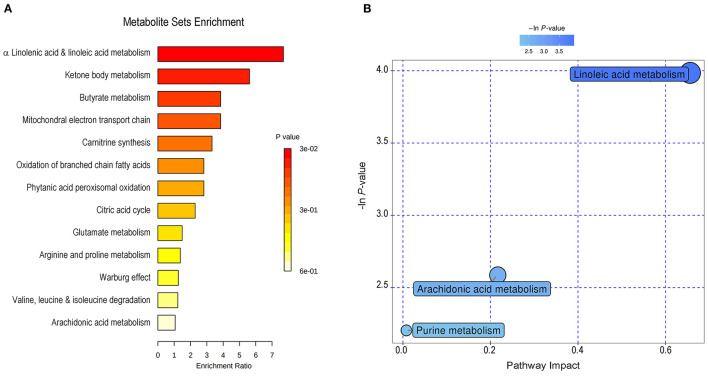
Metabolite set enrichment and pathway analysis of changed metabolites in SLK tear fluids. By MetaboAnalyst (http://www.metaboanalyst.ca), 50 significantly changed metabolites were subjected to metabolite set enrichment analysis **(A)** and to pathway analysis **(B)**. The main involved metabolic processes were noted with varying color according to the significance of each metabolite.

For the interpretation of differentially expressed metabolites and further investigation of the most significant metabolic pathways that may be involved in the pathophysiological mechanism of SLK, 50 significantly changed metabolites were subjected to set enrichment and pathway analysis. Figure 3 shows the metabolite set enrichment and metabolic pathways according to their biologically meaningful metabolite sets or pathway impact by selected databases. These changed metabolites are mainly involved in α linolenic acid and linoleic acid metabolism, ketone body metabolism, butyrate metabolism, mitochondrial electron transport chain, carnitine synthesis, oxidation of branched chain fatty acids, phytanic acid peroxisomal oxidation, citric acid cycle, and so on. The most significant pathway was linoleic acid metabolism. Both linoleic acid (LA), which has a high impact on the pathway, and eicosapentaenoic acid (EPA), another significantly altered metabolite, are associated with the biosynthesis and metabolism of the unsaturated fatty acid pathway. Notably, LA and EPA in the pathway were significantly decreased, indicating the potentially changed activity of the two metabolism pathways and their associated function in the process of SLK.

### Potential Biomarker Screening Associated With the ROC Curve

**Figure 4:**
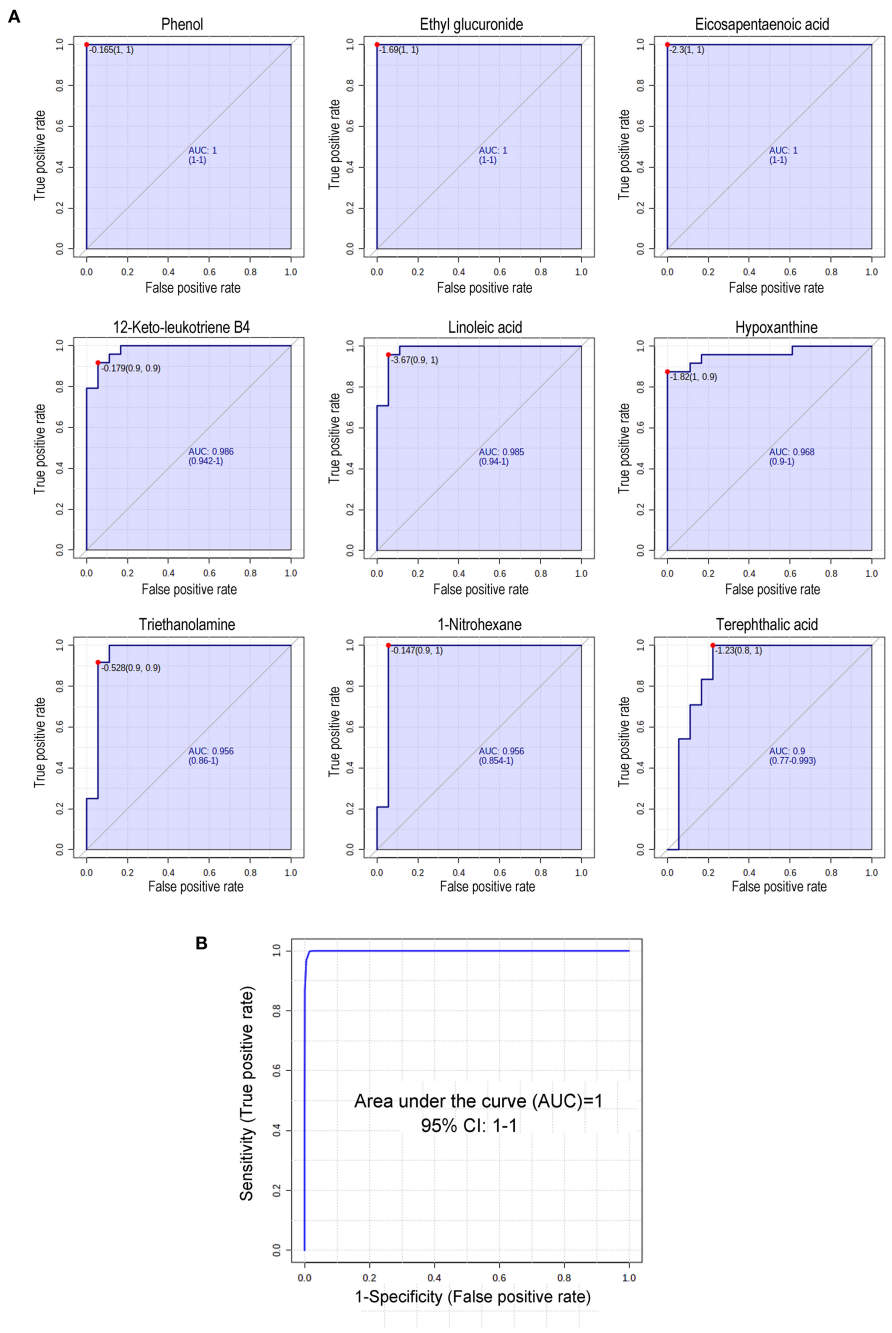
Receiver operating characteristic (ROC) curve analysis of 9 changed metabolites in SLK tear fluids. **(A)** Diagnostic efficacy evaluation using ROC curves of altered metabolites of tear between SLK and Ctrl individuals. For all 9 metabolites, the AUC ranged from 0.914 to 1 indicating good predictive ability. **(B)** According to data combining 9 metabolites in **(A)**, the cumulative ROC curve-based model evaluation (AUC = 1).

Receiver operating characteristic (ROC) curve analysis is generally considered to be the gold standard for the assessment of biomarker performance. Based on VIP, *P*-values, fold change, peak intensities and literature reviews, 9 metabolites were manually selected and identified as potential biomarkers capable of classifying SLK with high sensitivity (true-positive rate) and specificity (true-negative rate). As shown in Figure 4, the areas under the ROC curves (AUCs) for phenol, ethyl glucuronide, EPA, 12-keto-leukotriene B4, LA, hypoxanthine, triethanolamine, 1-nitrohexane, and terephthalic acid were 1, 1, 0.995, 0.986, 0.972, 0.972, 0.963, 0.958, and 0.914, respectively. Furthermore, an ROC curve-based biomarker model was established to evaluate the predictive power of the combined 9 metabolites. The cumulative ROC curve showed that the AUC value was 1, which gives the maximum confidence of differentiation and distinguishing SLK patients from Ctrl subjects. Moreover, the predicted class probability, after a 100-fold cross validation test, highlights 100.00% of samples correctly classified as SLK patients or as Ctrl subjects. Thus, one or more of these 9 metabolites can serve as potential biomarkers associated with SLK.

## Discussion

SLK is an ocular surface disorder of which the pathogenesis is not fully understood. Previous reports suggested that the underlying mechanical pathogenesis is likely a combination of mechanical injury, tear film instability, and inflammatory and autoimmune etiology (2) on the basis of investigations of patients' tear fluids or surgical removal of conjunctival tissues. For example, no galectin-3 expression in the abnormal conjunctiva areas of patients with SLK indicates a disturbance of mucosal epithelial barrier function, which may lead to tear film instability (27). Changed expression of stem cell factor, thymic stromal lymphopoietin, matrix metalloproteinases and tear cytokines, such as monocyte chemoattractant protein-1, shows the possible function of inflammation in SLK (5–7). Our present study demonstrated that the tear metabolic profiling of SLK patients was distinct from that of healthy controls, providing novel molecular insights into disease pathogenesis. Based on the union of VIP, *P*-value, and MS2 score, we confirmed 50 significantly different metabolites between the SLK and Ctrl groups. Again, metabolic pathways associated with these metabolites were probably involved in the development of SLK, of which low LA and EPA levels may play key roles on the basis of their highest impact.

Our data show that polyunsaturated fatty acid (PUFA)-related metabolites were changed in SLK patients. EPA, a ω-3 PUFA precursor, is the substrate of the production of 3-series prostaglandins and 5-series leukotrienes and have anti-inflammatory properties (28, 29). Furthermore, arachidonic acid (AA) derived from LA is an ω-6 PUFA and significantly decreased in our study (*p* < 0.001, data not shown because its MS2 score = 0.69). As a metabolic substrate for the generation of 2-series prostaglandin and 4-series leukotriene families (in the present study, 12-keto-leukotriene B4 in SLK/Ctrl = 0.368, *p* < 0.01) of eicosanoid mediators, the release of AA and the subsequent generation of eicosanoid lipid mediators are responsible for triggering inflammation under pathogenic conditions (13, 30, 31). Both ω-3 and ω-6 PUFAs modulate inflammatory processes by producing distinct classes of lipid mediators that play proinflammatory and proresolving functions. For example, resolvins, one of the four proresolving mediators, are synthesized from omega-3 (EPA and docosahexaenoic acid) and omega-6 (AA) fatty acids (32, 33). Current findings related to SLK suggest that an imbalance of ω-3 and ω-6 PUFAs, leading to an underproduction of proresolving lipid mediators, may promote non-resolving inflammation on the ocular surface, which is similar to findings in dry eyes (31, 34, 35). Studies in healthy human volunteers and in dry eye patients revealed that the effects of dietary supplementation with increasing ω-3 PUFA and ω-3 PUFA supplementation may rebalance the DHA/AA and EPA/AA ratios, leading to a change in the LTB5/LTB4 ratio in favor of less inflammation (36–38). However, PUFA effecting on SLK patients needs further study.

Apart from PUFA-related metabolites, our data includes other potential biomarkers for SLK *via* ROC curve analysis. However, it is difficult to clearly discuss the presence and role of these substances because the findings need further evaluation in this rare disease. Of the 9 metabolites, phenol and terephthalic acid, both involved in the aminobenzoate degradation pathway, increased significantly in SLK tear fluids, indicating their active functions in the metabolic regulatory network in the disease state. Phenol, 4-aminophenol, and terephthalic acid can be detected in blood, urine, or saliva by metabolomics analysis (39–45). In addition, phenol is one of the important components of volatile organic compounds that are generally considered to be toxic to the gut and are associated with ulcerative colitis, Crohn's disease, irritable bowel syndrome, celiac disease by metabolite profiling identification (46, 47).

Notably, our data showed that hypoxanthine decreased and adenosine increased in SLK tear fluids. These two substances are closely related in biosynthesis and degradation of the purine metabolism. Adenosine is an important neuroactive nucleoside and a homeostatic cellular modulator (48). It occurs in anoxic conditions and mainly produced by ATP breakdown. A study in rat brain extracts strongly suggests that intracellular ATP catabolism at normoxic concentration follows the pathway ATP⇄ADP⇄AMP → IMP → inosine⇄hypoxanthine. At ischemia/hypoxia concentration, intracellular ATP breakdown follows the pathway ATP⇄ADP⇄AMP → adenosine → inosine⇄hypoxanthine with little IMP formation (49). Recent studies have also demonstrated that the microenvironment at sites of inflammation often becomes profoundly hypoxic, due to a combination of increased oxygen demand and decreased supply (50–52). We are aware that cellular environment, metabolism, and inflammatory response are affected during ischemia/hypoxia. When considered together, whether the alterations of hypoxanthine and adenosine are related to SLK remains to be clarified.

In recent years, there have been a number of reports on tear metabolomics. However, due to different collection methods and various detection instruments, discrepancies are observed between reports. Our study had several limitations. First, considering the sensitivity of the instrument, we excluded some samples with little or too large tear volumes, which may result in sample selection bias. Second, our study did not further analyze the different metabolites according to the severity of SLK, which may provide disease-phase-related molecular information. In addition, it is important to emphasize that metabolomics data were obtained by a small group of tear samples of SLK and Ctrl individuals, and it would be necessary to confirm these data in studies with more patients and other tests.

In summary, we applied LC–MS/MS-based metabolomics to demonstrate the unique metabotypes of SLK and identified significant alterations in tear metabolites. The results obtained here not only provided potential diagnostic biomarkers for SLK screening but also expanded our understanding of the physiopathology of the disease. For the first time, metabolomic profiling revealed a possible change in the ω-3 and ω-6 PUFA balance in SLK patients, which demonstrates the inflammatory process in the pathogenesis of the disease. In addition, a combination model of 9 metabolites was identified as a potential biomarker in the present study to distinguish SLK patients from healthy controls, which should be validated in a larger and prospective cohort study.

## Data Availability Statement

The original contributions presented in the study are included in the article/Supplementary Materials, further inquiries can be directed to the corresponding author.

## Ethics Statement

The studies involving human participants were reviewed and approved by Human Research Ethics Committee of Zhongshan Ophthalmic Center. The patients/participants provided their written informed consent to participate in this study.

## Author Contributions

YZ identified patients, collected eye specimens, performed data analysis, and drafted the manuscript. CC identified patients, collected eye specimens, analyzed data, obtained ethical approval, and revised the manuscript. KL and RX identified patients and collected eye specimens. ZC revised the manuscript. XL organized control subjects and did sample treatment. KW supervised the study, participated in the discussions of each section of experiments, supported this research, and reviewed manuscript. All authors read and approved the final manuscript.

## Funding

This work was supported by grants from the National Natural Science Foundation of China (81770896, 81970848) and the Guangzhou Science Technology and Innovation Commission (201607020011).

## Conflict of Interest

The authors declare that the research was conducted in the absence of any commercial or financial relationships that could be construed as a potential conflict of interest.

## Publisher's Note

All claims expressed in this article are solely those of the authors and do not necessarily represent those of their affiliated organizations, or those of the publisher, the editors and the reviewers. Any product that may be evaluated in this article, or claim that may be made by its manufacturer, is not guaranteed or endorsed by the publisher.
