# Supplementary figures and images for: Metabolomic Alterations in the Tear Fluids of Patients With Superior Limbic Keratoconjunctivitis
Source: Front Med (Lausanne). 2022 Jan 18;8:797630. doi: 10.3389/fmed.2021.797630 (PMC8804220; doi:10.3389/fmed.2021.797630)

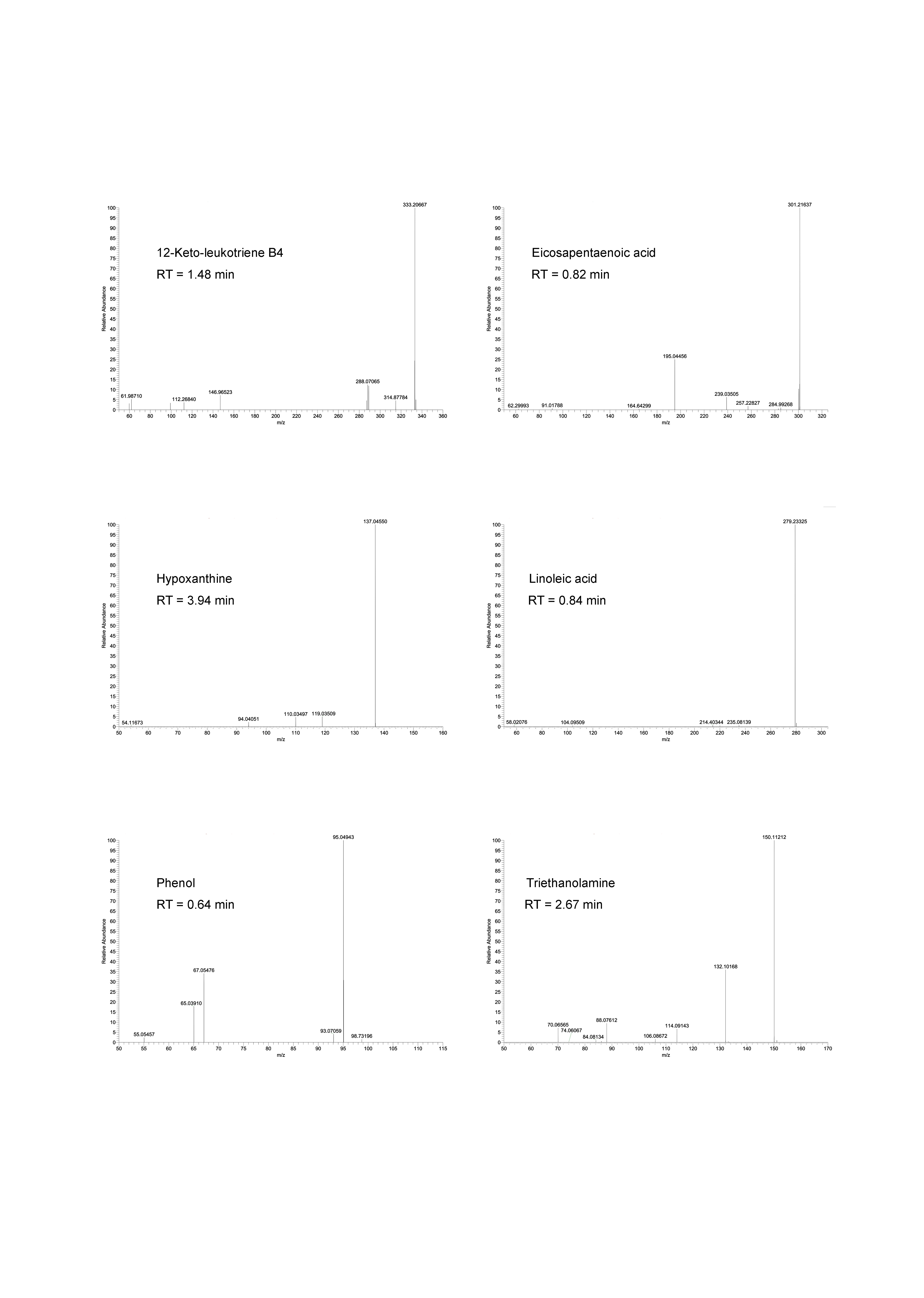

Supplement: Supplementary file 1 [file Image_1.JPEG]
